# *QuickStats:* Age-Adjusted Death Rates* for Female Breast Cancer,^†^ by State — National Vital Statistics System, United States, 2019^§^

**DOI:** 10.15585/mmwr.mm7039a6

**Published:** 2021-10-01

**Figure:**
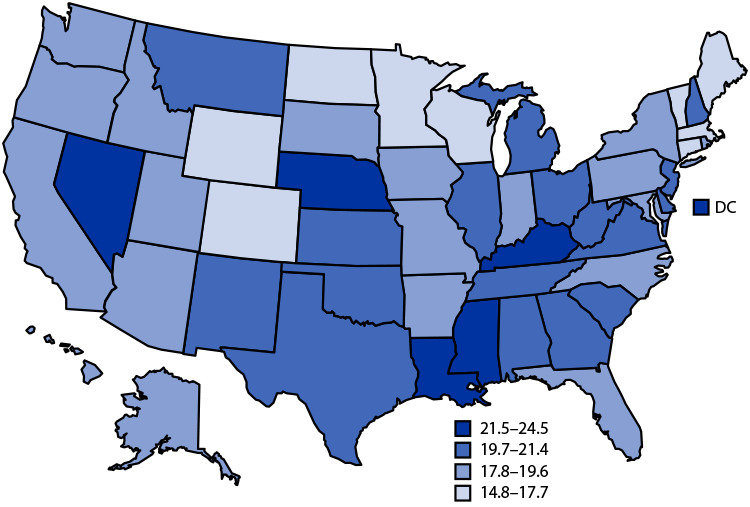
In 2019, the age-adjusted rate of female breast cancer deaths in the United States was 19.4 per 100,000 population. Jurisdictions in the highest category for breast cancer death rates were DC (24.5), Nevada (23.7), Nebraska (22.4), Kentucky (22.2), Louisiana (22.0), and Mississippi (22.0). Those in the lowest category were North Dakota (14.8), Massachusetts (15.3), Vermont (16.2), Connecticut (16.8), Wyoming (17.2), Minnesota (17.5), Colorado (17.6), Wisconsin (17.6), and Maine (17.7).

**Source:** National Vital Statistics System, Mortality, 2019. https://www.cdc.gov/nchs/nvss/deaths.htm

